# Preventing profiling for ethical fake news detection

**DOI:** 10.1016/j.ipm.2022.103206

**Published:** 2023-03

**Authors:** Liesbeth Allein, Marie-Francine Moens, Domenico Perrotta

**Affiliations:** a European Commission, Joint Research Centre (JRC), Italy; b Department of Computer Science, KU Leuven, Belgium

**Keywords:** Fake news detection, Ethics, Profiling, Natural language processing, Constrained representation learning

## Abstract

A news article’s online audience provides useful insights about the article’s identity. However, fake news classifiers using such information risk relying on profiling. In response to the rising demand for ethical AI, we present a profiling-avoiding algorithm that leverages Twitter users during model optimisation while excluding them when an article’s veracity is evaluated. For this, we take inspiration from the social sciences and introduce two objective functions that maximise correlation between the article and its spreaders, and among those spreaders. We applied our profiling-avoiding algorithm to three popular neural classifiers and obtained results on fake news data discussing a variety of news topics. The positive impact on prediction performance demonstrates the soundness of the proposed objective functions to integrate social context in text-based classifiers. Moreover, statistical visualisation and dimension reduction techniques show that the user-inspired classifiers better discriminate between unseen fake and true news in their latent spaces. Our study serves as a stepping stone to resolve the underexplored issue of profiling-dependent decision-making in user-informed fake news detection.

## Highlights

• Ethical fake news detection in terms of user data integration.
• Profiling-based decision-making is prevented while leveraging social knowledge.
• Social science-based losses integrate social context during model optimisation.
• The losses assume identity correlations between news texts and Twitter spreaders.
• Context-inspired classifiers learn statistically better discriminating latent spaces.

## 1 Introduction

Disinformation – often called *fake news* – is not new (Allcott and Gentzkow, 2017, Tandoc et al., 2018). People have been creating and spreading intentionally inaccurate and misleading stories since we started living in hierarchical communities (Burkhardt, 2017). From political smearing campaigns in Ancient Rome to war and propaganda campaigns during the First and Second World War (Posetti & Matthews, 2018), people have used disinformation to deceive and persuade others. Nowadays, low-resource online platforms such as social media and websites allow us to spread deceitful information at an unprecedentedly high rate and large scale. Key actors in this kind of dissemination are the authors who create deceptive content (= *creators*) and the platform users who subsequently spread it (= *spreaders*). As creators are often spreaders as well, we refer to both actors as *users*. Although researchers have been exploring automated disinformation detectors that take both textual content and user information as input, existing approaches risk relying on *profiling*. *Following the rising demand for ethical AI, this paper designs, implements, and analyses a profiling-avoiding learning algorithm that introduces user information from Twitter to text-based classifiers predicting the veracity of a given online news article in a more ethical, indirect manner*.

### Decision-making on profiling should be avoided.

According to the General Data Protection Regulation (GDPR) of the European Parliament and Council (European Union, 2016), people have the right not to be subject to decisions based on automated, ‘human-out-of-the-loop’ processing that assesses their personal aspects — especially when those decisions produce legal effects concerning them. The GDPR also explicitly discusses profiling.^1^ Profiling can be used to describe all kinds of automated processing of personal data that make decisions based on analyses or predictions of personal aspects such as behaviour, interests, and reliability. Profiling is allowed under a few conditions, but should always safeguard a person’s integrity. For example, the risk of errors should be minimised and decision-making should refrain from discriminating people on the basis of personal aspects such as religion, sexual preference, and political views (European Union, 2016). In this work, *we prevent decision-making based on profiling by removing explicit user data from the decision process while inspiring a classifier’s parameters with user insights during training.*

### Profiling-avoiding detection models still need people.

We focus on a specific type of disinformation, namely fake news - a politically-loaded concept that has been popularised since the 2016 US Presidential elections (Cunha, Magno, Caetano, Teixeira, & Almeida, 2018). Fake news is used to describe a qualitative property of information that is presented as legitimate news but intentionally violates its function of conveying truthful information to the public (Brown, 2019). In other words, fake news are seemingly legitimate news articles that contain intentionally inaccurate or misleading content. Although satire ticks all the boxes, it fulfils entertainment purposes whereas fake news creators are commonly driven by ideological and/or financial gains. For example, they want to change a reader’s beliefs (i.e., ideological) or receive higher ad revenues from more clicks (i.e., financial). The stress on intention and motivation spawns a distinction between intentionally and accidentally false information, establishing a difference in fake and false information, or respectively, disinformation and misinformation (Bontcheva, et al., 2020, Brown, 2019). We should thus regard those who create and spread fake news as fake news creators and fake news spreaders if – and only if – (a) they are aware of the falsity and inaccuracy of the information they create and spread; (b) they intend to mislead or deceive their audience; and (c) they are ideologically and/or financially motivated. As a result, correctly identifying fake news on both content and creator/spreader level is extremely challenging and requires an in-depth understanding of a person’s knowledge, intentions, and motivations.

### Do not let models use people, but let people inspire models.

**Fig. 1:**
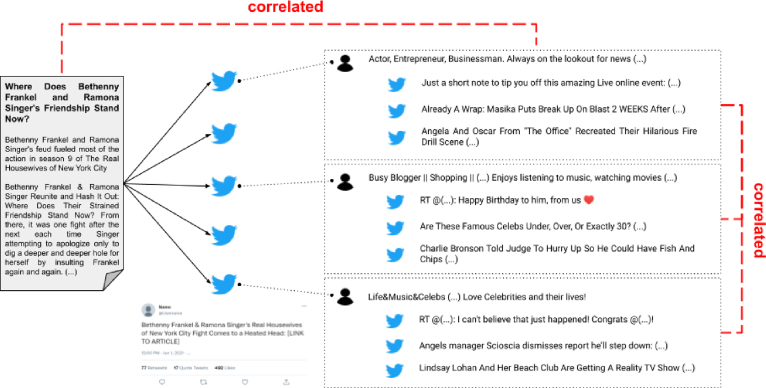
Illustration of a ‘true’ news article spread by a number of tweets. This work introduces the *correlated identity assumption*, which argues that (a) the identity of the article (reflected in its content) and the identity of its spreaders (reflected in their profile description and tweets) are correlated; and (b) the identities of its spreaders correlate too. During the training phase of a fake news classifier, the two correlations are enforced by a cross-modal loss function. For privacy reasons, the profile descriptions and tweets displayed are combined representations of several profiles.

**Fig. 2:**
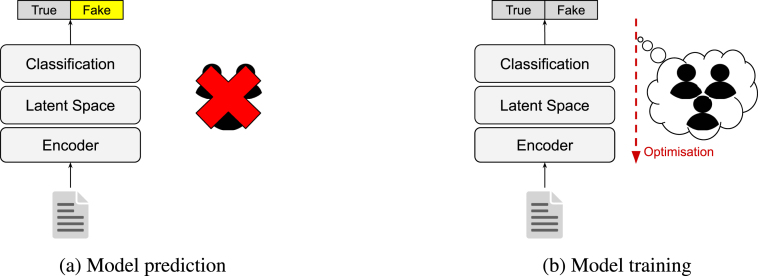
Our approach to ethical, user-inspired fake news detection. During veracity prediction (a), a model predicts whether or not a given news article contains disinformation — without access to any user information. During training (b), user information is introduced to the model in the optimisation step.

As fake news is strongly linked to human intentions, we find it essential to present fake news detectors – either human or computational – with the human actors involved in its dissemination on social media. The detectors should then be able to better situate an online news article in its social context. Instead of simply presenting the users as model input and risking model decisions on profiling, we design a multimodal learning algorithm that hides user information during prediction but integrates user insights during training. For this, we take inspiration from the social sciences and formulate a *correlated identity assumption*. This novel assumption argues that the identity of a news article and that of its spreaders are somehow correlated (Fig. 1). We formalise the assumption as a cross-modal objective function that computes the distance between the latent representation of the article and each spreader, and between the latent representations of all spreaders of the same article. During optimisation, the objective function is minimised so that cross-modal correlations are introduced in the parameters of a text-based classifier. The learning algorithm, as it were, *inspires* the model with social context and user insights during optimisation but the model cannot directly *use* that user information in its decision process (Fig. 2). Our study is the first to design, implement, and analyse a learning algorithm that elegantly tackles the underexplored problem of profiling-dependent fake news detection using a cross-modal loss function from the social sciences.

### Contributions.

We summarise the main contributions of this work as follows:

Taken from GossipCop (Shu, Mahudeswaran, Wang, Lee, & Liu, 2020).

• *Novel approach to ethical fake news detection.* Instead of focusing on explainability and bias mitigation as done by the majority of work on ethical fake news detection, we tackle the issue of decision-making on profiling and challenge the way user data is integrated in fake news classifiers.
• *From user-reliant and profiling-dependent to user-inspired and profiling-avoiding fake news detection.* Our learning algorithm excludes the user modality as explicit model input but integrates user knowledge during model optimisation. This way, decision-making on profiling is prevented while the classifier can indirectly exploit the rich insights the social context of news articles holds.
• *Encoding of article and users in isolation with fusion via loss functions.* While an article encoder encodes the content of an article as part of a classifier’s decision-making process, an isolate encoder represents Twitter users in the same latent space as the articles. During model optimisation, a cross-modal loss function takes the latent representations of the article and its spreaders, and enforces maximised correlation between the two. We are the first to design, implement, and evaluate this kind of user integration method for fake news detection.

### Structure of the article.

Section 2 presents related work on ethical and multimodal fake news detection; Section 3 formally defines the task and describes the user-inspired learning algorithm, the various model architectures, and the cross-modal loss function; Section 4 states the problem and introduces the research questions; Section 5 presents the data, experimental setup, and results; Section 6 discusses the experiments and briefly covers the ethics surrounding fake news detection; Section 7 elaborates on the implications of this work; finally, Section 8 concludes the article.

## 2 Related work

### Ethical fake news detection.

Computational research that (in)directly tackles the ethical side of fake news detection and related tasks, such as fact-checking and offence detection, is mainly limited to explainability and bias mitigation. Regarding explainability, Ahmadi, Lee, Papotti, and Saeed (2019) defined a number of rules to extract facts from knowledge graphs that later served as explanations. Reis, Correia, Murai, Veloso, and Benevenuto (2019) explained a fake news detection model’s predictions by returning the most important input features using Shapley additive explanations, while Chien, Yang, and Yu (2022) returned post-hoc explanations displaying the individual contributions of content, user, and sentiment features to a news article’s predicted veracity. Lu and Li (2020) visualised attention weights to highlight suspicious retweeters and language. On a more interactive level, Zhang, Rudra, and Anand (2021) developed a fact-checking tool that presented users with a veracity decision and supporting and/or refuting evidence sources found on Wikipedia. The sentences or sentence parts supporting the decision were highlighted, and users were asked to mark whether they agreed or disagreed with the highlighted evidence. Instead of relying on the weights of a model for explanation, Atanasova, Simonsen, Lioma, and Augenstein (2020), Kotonya and Toni (2020) and Kazemi, Li, Pérez-Rosas, and Mihalcea (2021) applied extractive and abstractive summarisation techniques to provide users with justifications in natural language. Regarding bias mitigation, several mitigation methods have been proposed (Hovy & Prabhumoye, 2021). For example, Murayama, Wakamiya, and Aramaki (2021) explicitly masked person names in fake news detection models to tackle diachronic biases found in time-limited datasets. Binns, Veale, Van Kleek, and Shadbolt (2017) discussed normative bias and investigated how different norms about offensive speech among the data annotators can bias automated content moderation models. Al Kuwatly, Wich, and Groh (2020) identified annotator bias based on several demographic characteristics such as age, first language, and education level that leads to biased abusive language and hate speech detectors. Lastly, Wich, Bauer, and Groh (2020) found a negative effect of political bias in hate speech detection models and later developed a framework to analyse and uncover inherent biases in abusive language datasets (Wich, Eder, Al Kuwatly, & Groh, 2022). In this paper, we address the ethical principles of fairness and prevention of harm (High-Level Expert Group on AI, 2019). According to those principles, an ethical AI system should not discriminate or stigmatise any group or individual, and a person’s integrity needs to be respected. In contrast to previous work, we mitigate violations of those principles not by generating explanations or mitigating bias but by adapting the integration and fusion mechanisms of information.

### Fake news detection: more than just text.

In fake news detection and related tasks, input beyond the text of a message has been used to predict a message’s reliability, veracity, or harm. Some focused on predicting the veracity of images (Gupta et al., 2013, Zlatkova et al., 2019) or took images as supporting input to multimodal models detecting fake news in texts (Jin, et al., 2017, Nakamura et al., 2020, Wang, et al., 2018). Soldner, Pérez-Rosas, and Mihalcea (2019), for example, detected deceptive utterances in dialogues using text and video.  Chen, et al. (2022) considered both text and image as input and quantified their ambiguity by computing cross-modal correlations. Others moved beyond text and image, and leveraged contextual features on propagation (Bian, et al., 2020, Zhou and Zafarani, 2019), source (Yuan, Ma, Zhou, Han, & Hu, 2020), and time (Allein et al., 2021, Song, Shu, and Wu, 2021). Shu, Mahudeswaran, Wang, and Liu (2020), for example, combined multiple contextual features in multimodal, hierarchical propagation networks using linguistic, structural, and temporal features from micro-level and macro-level propagation networks to detect fake news on Twitter. More recently, Sheng, et al. (2022) constructed for each target news item a time-constrained news environment of both related and unrelated news items, which indicates the perceived popularity and novelty of the target news item. As this paper focuses on integrating user information in text-based fake news detection models, we continue to discuss the fake news detection literature that leveraged users.

### Leveraging users for fake news detection.

Some fake news detection tasks revolve around users. Automated models have been developed that predict a user’s tendency to spread fake news (Rangel et al., 2020, Sansonetti et al., 2020) or rumours (Sharma & Sharma, 2021). Ferrara, Varol, Davis, Menczer, and Flammini (2016) looked at the individuals or organisations behind Twitter profiles and tried to detect social bots. In those tasks, users were mainly represented by profile metadata such as number of followers, often in combination with linguistic properties and personality traits that were extracted from their tweets (Balestrucci and De Nicola, 2020, Giachanou et al., 2021). Recently, Zhou, Shu, Phoha, Liu, and Zafarani (2022) modelled the intent of fake news spreaders and demonstrated its usefulness when detecting fake news.

Instead of entirely focusing on the users, fake news detection models have been taking social media users as additional input to detect fake messages online. Kim, Tabibian, Oh, Schölkopf, and Gomez-Rodriguez (2018) and Ruchansky, Seo, and Liu (2017), for example, represented a user by their interactions with true and fake content. Kim, et al. (2018) constructed for each user a structured tuple containing that user’s ID, the time at which the interaction occurred, and the ID of the article. Ruchansky et al. (2017) enriched such tuples by replacing the user ID with a binary incidence matrix that indicated for all articles in the dataset whether the user had engaged with them or not. As a result, users who share common interests are represented similarly, and the interaction between them could be derived by comparing their representations. That kind of approach, however, is heavily dataset-dependent as it merely represents the user by their interactions with a closed set of articles. The approach also refrains from explicitly representing the interactions and relations between the users. By contrast, our algorithm leverages user information that is not directly related to the news articles in the dataset and exceeds representations of one-way interactions.

Some works took into account richer user information and described the nature of the interaction between a user and an article. Qian et al., 2018, Shu, et al., 2019, Zhang et al., 2019; and Shu, Mosallanezhad, and Liu (2022) leveraged comments and/or replies to a news article or tweet. Song, Yang, et al. (2021) also took into account the retweets that commented on an original tweet. Such user representations reflect a user’s opinion and stance towards a tweet or news article. However, the approach is restricted by the brevity of the comments. Moreover, the user representations are situation-specific as they are constructed using a comment or reaction to a single message. By contrast, we construct user representations that exceed comments to tweets and retweets. In our algorithm, each user is represented by their profile description and/or their own tweets. The algorithm thus relies on user-generated content that is not exclusively related to the message that is evaluated.

Other works focused on modelling users and their interactions on a more in-depth level to support fake news detection. A popular approach is to construct heterogeneous graphs of article and user nodes where articles share edges with users who interacted with them, and where users are linked to users with whom they share explicit social relations like followership  (Chandra, et al., 2020, Min, et al., 2022, Nguyen et al., 2020). It could be argued that meaningful social groups can be formed from the users’ social networks. Nonetheless, we do not leverage this kind of information as such groups do not necessarily contain users that share common interests. According to Fani, Bagheri, and Du (2017), a follower-following connection can simply indicate kinship. Twitter also differs from other social media platforms in terms of the reciprocity of user connections (Kwak, Lee, Park, & Moon, 2010). Instead of networking, Twitter users mainly reside to the platform to obtain information and news. This goal is reflected in the low number of mutual follower-following relations between the users (Kwak et al., 2010). We therefore model the relations between users in terms of their common interest rather than their explicit connection on Twitter. This rationale is supported by Mehta, Pacheco, and Goldwasser (2022), who connected users with similar news interactions. To obtain interest-based social groups, we look at the articles the users have shared and group users who shared the same article. Our learning algorithm will then force a text-based fake news classifier to reason about their correlations.

## 3 Methodology

We start by formally defining the fake news detection task (Section 3.1). We then conceptualise the multimodal, profiling-avoiding learning algorithm and the assumption it is based on, and motivate the algorithm against alternative profiling mitigation approaches (Section 3.2). Next, we present the text encoding architectures used for modelling news articles and users (Section 3.3), and describe the loss functions that form the basis for the optimisation process (Section 3.4).

### 3.1 Formal task definition

We approach fake news detection as a binary classification task: fake news classifier f=c∘h, with h a neural text encoder and c a single linear classification layer with softmax activation, predicts whether a given news article a is *true* (y=1) or *fake* (y=0).

(1) $$h:a\mapsto a^{\prime },a^{\prime }\in R^{m}\;\;c:a^{\prime }\mapsto y,y\in \left\{0,1\right\}$$

During training, user encoder g models users u who spread a on Twitter, and projects them onto the same latent space as a. As u is represented by text, g is a neural text encoder. The learning algorithm then leverages g(u), h(a), and y for optimising f, where g(u) will only be used in the loss function. A detailed description of the algorithm is given in the coming sections.

(2) $$g:u\mapsto u^{\prime },u^{\prime }\in R^{m}$$

In sum, the following textual data is used (see Table 1):

• $A=\left\{\left(a_{i},y_{i}\right)|0<i\leq N\right\}$ is the set of N **online news articles** in the training set. Each news article $a_{i}$ is represented as a concatenation of its title $t_{i}$ and its body text $b_{i}$: $a_{i}=\left[t_{i};b_{i}\right]$. The ground-truth label $y_{i}\in \left\{0,1\right\}$ indicates whether $a_{i}$ is fake ($y_{i}=0$) or true ($y_{i}=1$).
• $U=\left\{u_{j}|0<j\leq K\right\}$ is the set of K **Twitter users** who spread one or more articles in A. Depending on the user setup (see Section 5.2), user $u_{j}$ is represented by their profile description $d_{j}$, their tweet timeline ${tw}_{j}$, or a concatenation of both ($u_{j}=\left[d_{j};{tw}_{j}\right]$).
• $U_{i}\subseteq U$ is the **subset of users who shared article** $a_{i}$. Each user subset $U_{i}$ consists of S users $u_{j}$, with S≤10.^2^ We automatically obtain $U_{i}$. The datasets (see Section 5.1) paired each $a_{i}\in A$ with a list of tweet IDs that shared a link to $a_{i}$. Starting with the lowest tweet ID, we extract the user IDs given in the metadata of the tweets until we reach S user IDs. Finally, each user behind the user IDs is transformed to its user representation, and all S user representations are combined in $U_{i}$. The lower the tweet ID, the older the tweet and the closer in time it is to the first tweet that introduced $a_{i}$ on Twitter. This way, $U_{i}$ contains the first, still retrievable S users in the Twitter dissemination process of $a_{i}$. As $U_{i}$ is automatically obtained, $u_{j}$ can be part of multiple user subsets when that user has shared more than one $a_{i}\in A$.

We appreciate the non-binary nature of fake news but opt for this classification approach for data availability reasons. This approach also allows us to more intuitively analyse the impact of our user-inspired learning algorithm on detection performance. Although there exist datasets that have more differentiated labels, they lack the information we need for our approach, such as spreader information (Augenstein, et al., 2019, Silverman, et al., 2016, Song, Petrak, et al., 2021) or full news articles (Mitra and Gilbert, 2015, Zubiaga, et al., 2016).

**Table 1:** Overview of the features used to represent a news article ($a_{i}$) and a user who spread the article on Twitter ($u_{j}\in U_{i}$). Article $a_{i}$ is represented as a concatenation of $t_{i}$ and $b_{i}$: $\left[t_{i};b_{i}\right]$. Depending on the user setting, user $u_{j}$ is represented as $d_{j}$, $tw_{j}$ or $\left[d_{j};tw_{j}\right]$.

News article representation: $a_{i}$	

$t_{i}$	Title
$b_{i}$	Body text

User representation: $u_{j}\in U_{i}$	

$d_{j}$	Profile description
$tw_{j}$	User timeline containing max. 200 latest tweets

### 3.2 A user-inspired learning approach

Social media users are important actors in an article’s dissemination online and provide valuable insights into its spreaders. A closer look at the spreaders individually and as a group might reveal commonalities between the individuals and the news article. This kind of information could then help a fake news detection model to contextualise the article’s appeal. In order to leverage such insights within a model, we delve into the social science literature to find out how a news article and, by extension, its creator relate to their audience/spreaders on social media.

#### Correlated identity assumption.

Creators write news articles for several reasons: they might want to entertain, inform, or – in the case of fake news – deceive their readers. Likewise, spreaders have their own reasons for disseminating articles. They do not necessarily align with those of the creators. For example, a spreader who is unaware of an article’s intentional deceptiveness might share it because the spreader finds its subject entertaining. Irregardless of why someone creates or shares articles, those articles attribute to that person’s online social identity (Marwick & Boyd, 2011). The identity-driven motivations for creating and spreading information can be divided into two types (Marwick & Boyd, 2011):

(a) *Audience-oriented* motivations
(b) *Self-oriented* motivations

**Fig. 3:**
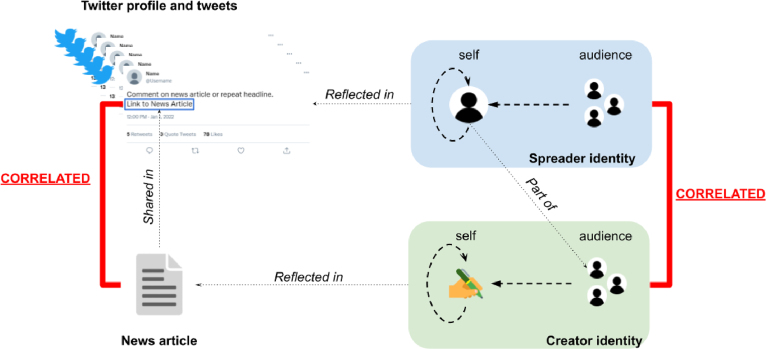
Illustration of the correlated identity assumption. Both the identity of the spreader (in blue) and the creator (in green) are constructed by self-oriented and audience-oriented motivations (dashed arrows). The spreader identity and creator identity are reflected in the twitter profile/tweets and the news article, respectively. The spreader who shares a link to the news article is part of the creator’s audience and should thus indirectly correlate with the creator’s identity. As a result, we assume that not only the spreader identity and the creator identity but also the twitter profile/tweets and the news article should be somehow correlated (in red) as they are reflections of their identities . (For interpretation of the references to colour in this figure legend, the reader is referred to the web version of this article.)

When both creators and spreaders share information, they target an ideal audience from whom they want to attract certain responses (*audience-oriented*). Those responses could be visible on the online platform on which the information was presented, e.g., reactions, retweets or likes, or could be more personal, e.g., change in opinion or stronger interpersonal connection. Fake news creators intentionally deceive and persuade readers. The content and style of fake news articles reflect such audience-oriented motivations: they contain emotion-provoking words, discuss controversial topics, and are guided by persuasion rhetorics (Przybyla, 2020, Shu, et al., 2017). Spreaders, however, do not necessarily share the same audience-oriented motivations as the creators when they spread a fake news article. Some just want to inform or entertain their Twitter followers, while others debunk the fake information in the article (Giachanou, Ríssola, Ghanem, Crestani, & Rosso, 2020). Even though the audience-oriented motivations that drive creators and spreaders may differ, the content they decide to create and spread attributes to the online identity they want to portray (*self-oriented*). That online identity reflects the meta-image of the self and is part of an individual’s self-commodification or personal branding (Khamis et al., 2017, Marwick and Boyd, 2011). We presume that a person’s social identity online results from the interplay between audience-oriented and self-oriented motivations. For example, a spreader might choose to share a news article about a specific celebrity because the spreader is a fan (*self-oriented*), wishes to portray themself as such towards others in their online social network (*self-oriented* and *audience-oriented*), and wants to entertain their network (*audience-oriented*). In the case of Twitter, a user’s tweet collection, profile description, and shared content arguably reflect that user’s online identity. Assuming that users do not portray disparate identities within one profile, we presume that a user’s identity contained within his/her tweets and profile description (i.e., user-created content) correlates with the identity of the news article (i.e., user-shared content). Furthermore, such correlations should be found across all created and shared content — independent of the topic they discuss. If we assume that a news article correlates to some extent to each of its spreaders, we can also argue that the spreaders should show some commonalities among each other as they are part of the article’s audience. We combine the assumed correlations between the users and the article, and between the users themselves in the *correlated identity assumption* (Fig. 3).

**Figure:**
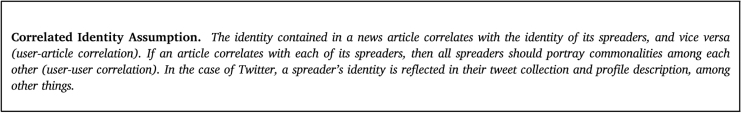

#### Multimodal learning algorithm.

**Fig. 4:**
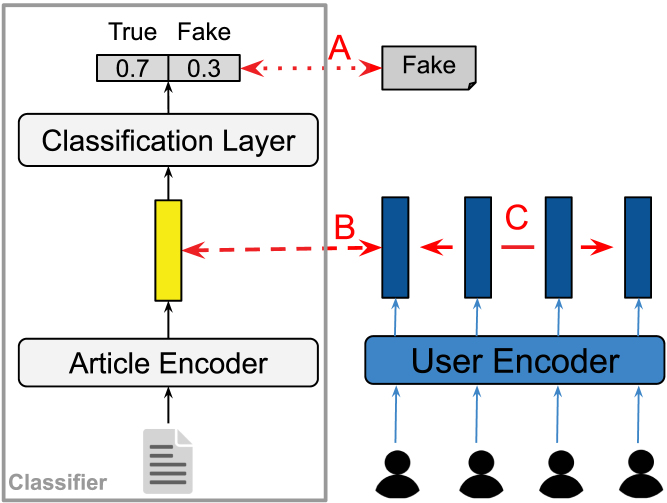
Overview of the multimodal learning algorithm. A classifier predicts the veracity of a given article. In parallel, a user encoder encodes a subset of users who shared that article on Twitter. During training, the algorithm optimises all parameters on three learning objectives (in red): (A) discriminate between fake and true news; (B) correlate article and each user; and (C) correlate all users in the subset . (For interpretation of the references to colour in this figure legend, the reader is referred to the web version of this article.)

The multimodal learning algorithm relies on the correlated identity assumption to incorporate user insights in a text-based fake news classifier. Firstly, the assumption argues that a user’s identity correlates with the identity of the article (s)he shares (i.e., user–article correlation ρ): $\forall u_{j}\in U_{i}\wedge a_{i}\Rightarrow \rho \left(a_{i},u_{j}\right)$. However, it is not straightforward to pinpoint how those correlations exactly look like. Instead of reasoning about possible correlations ourselves, the learning algorithm forces classifier f=c∘h to look for them on its own. It does this by enforcing a correlation objective function on the model’s latent presentation of the article, $h\left(a_{i}\right)$, and the latent representation of the users, $g\left(u_{j}\right)$, encoded by a separate user encoder g. The correlation between $h\left(a_{i}\right)$ and $g\left(u_{j}\right)$ should then be maximised during training. Secondly, the spreaders of a single article are assumed to portray some commonalities too (i.e., user–user correlation ρ): $\forall u_{j}\in U_{i}\wedge u_{k\neq j}\in U_{i}\Rightarrow \rho \left(u_{j},u_{k}\right)$. Again, these correlations are hard to define. Similar to the article–user correlations, the model needs to find the user–user correlations on its own. The learning algorithm therefore enforces a second correlation objective function that takes $U_{i}$ and computes the correlation between the encoded representation of each individual user $g\left(u_{j}\right)$ and the encoded representation $g\left(u_{k}\right)$ of each other user in $U_{i}\setminus u_{j}$. As the fake news classifier needs to predict a news article’s veracity as flawlessly as possible, the learning algorithm’s third objective is to reduce the classifier’s prediction error. For this common classification objective, the algorithm takes the model’s yielded probability distribution over all prediction labels and the ground-truth label, and minimises the prediction error during optimisation. For sake of simplicity, we transform the two correlation objective functions to loss functions so that all three objective functions can be minimised. So instead of correlation maximisation, the distance between the latent representations is minimised. In all, the multimodal learning algorithm consists of three training objectives that are each supported by a loss function (Fig. 4): (a) minimise the prediction error using a prediction loss (i.e., $L_{pred}$); (b) correlate the article and the users using a distance loss (i.e., $L_{dist\left(\alpha \right)}$); and (c) correlate the users using a distance loss (i.e., $L_{dist\left(\beta \right)}$). The loss functions are formulated in Section 3.4.

#### Motivation over other profiling mitigation methods.

Intuitive approaches to curb profiling in fake news classifiers would be to either minimise the impact of user profiling or control user modelling. Let us take classifiers l and m, which are any type of classifier taking users u as only input and additional input to news article a, respectively:

(3) l:u↦y,y∈{0,1}

(4) m:a,u↦y,y∈{0,1}

Given that u is its only input, it is virtually impossible for l to minimise the impact of profiling. In the case of m, lower importance could be assigned to those parameters responsible for modelling u while those modelling a receive higher importance. Nonetheless, this approach merely reduces the effect of profiling on model predictions. It does not avoid or prevent it. Profiling avoidance, in this case, would entail ignoring u when predicting y, which boils down to m being a text classifier that is unaware of any social context surrounding a. Following the definition of profiling as “all kinds of automated processing of personal data that make decisions based on analyses or predictions of *personal aspects*”, another approach would entail removing information signalling *personal aspects* from the representation of u at any stage within the model. However, such signals are deeply entangled within and across various profile, network, and content features of u. It is thus difficult to meticulously change, hide, or remove all of them. In this work, we do not reduce the impact of u in a classifier or attempt to alter the representation of u to avoid profiling. We instead remove u as model input completely so that predictions can only be based on a. Since u carries useful insights into the social context of a, u is indirectly integrated in the classifier’s parameters via a cross-modal loss function L during model optimisation.

### 3.3 Text encoding

To explore the impact of our multimodal learning algorithm on various architectures for h and g, we experiment with three text encoding architectures that encode $a_{i}$ to $h\left(a_{i}\right)$ and $u_{j}$ to $g\left(u_{j}\right)$: CNN, HAN, and DistilBERT. As $a_{i}$ and $u_{j}$ are both represented using textual data only, we apply the same preprocessing to $a_{i}$ and $u_{j}$, and h and g adopt the same encoding architecture when modelling $a_{i}$ and $u_{j}$, respectively. The neural encoding architectures have been extensively used as bases for detection models in the fake news literature (Mridha, Keya, Hamid, Monowar, & Rahman, 2021). For sake of simplicity, we name each fake news classifier f by the encoding architecture for h and g. We illustrate the neural architectures using $a_{i}$ and h but note that the same preprocessing and modelling is adopted for $u_{j}$ and g.

**CNN**. We follow the approach of Kim (2014). A word embedding layer takes article $a_{i}$, with $\left|a_{i}\right|\leq 500$, and transforms each token to its 300-dimensional pretrained GloVe embedding.^3^ Next, three parallel convolutional layers with ReLU activation and (3, 4, 5) filter windows each encode the article’s word embedding representation to a 100-dimensional latent representation. The model then concatenates those three latent representations, resulting in a 300-dimensional latent representation of the article, $h\left(a_{i}\right)$.

**HAN**. We adopt the approach of Yang, et al. (2016). In contrast to the CNN model, the Hierarchical Attention Network (HAN) encodes article $a_{i}$ on two levels, namely word and sentence level. Before feeding $a_{i}$ to the model, we need to additionally split the article into sentences^4^ using the sentence tokenizer from the NLTK toolkit (Bird et al., 2009). So instead of a one-dimensional vector, $a_{i}$ is a two-dimensional Z×T matrix containing Z sentences with T tokens per sentence. We set Z=50 and T=50. As done in the CNN model, a word embedding layer transforms each token in $a_{i}$ to its 300-dimensional pretrained GloVe embedding. A word-level encoding layer then encodes Z 100-dimensional latent sentence representations using a bidirectional GRU with word-level attention. The Z sentence representations are then fed to a bidirectional GRU with sentence-level attention. The sentence-level encoder ultimately yields a 100-dimensional latent representation of the article, $h\left(a_{i}\right)$. Both the word-level and sentence-level bidirectional GRU have hidden size = 50 and return their concatenated last hidden state as sentence representation and article representation, respectively.

**DistilBERT**. We take a pretrained, Transformer-based DistilBERT model (Sanh, Debut, Chaumond, & Wolf, 2019) as article encoder.^5^ Its architecture is based on the BERT model (Devlin, Chang, Lee, & Toutanova, 2019) but the model serves as a faster alternative with fewer parameters. It contains an embedding layer and five Transformer blocks with multihead attention and takes textual input up to 512 tokens. The DistilBERT model takes article $a_{i}$ and returns a 768-dimensional latent representation of the article, $h\left(a_{i}\right)$, by max-pooling the hidden states of its last encoding layer.

**Fig. 5:**
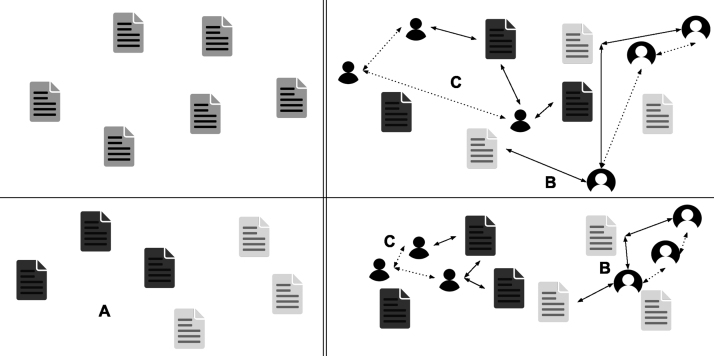
Illustration of the three training objectives of the multimodal learning algorithm. The first objective (A) is a common classification objective (*left*): the classifier should successfully discriminate between fake and true news. To leverage social context in the text-based classifier, the algorithm projects the users who shared at least one of the articles in the same latent space as the articles (*right*). The second objective (B) then forces the model to project an article close to the users who spread it by looking at their correlation. Similarly, the third objective (C) groups users sharing the same article in the latent space so that people with similar interests are projected closely to each other.

### 3.4 Loss functions

In the multimodal learning algorithm, the parameters of classifier f=c∘h are optimised using three objectives (Fig. 5). The first objective is a classic classification objective where a model should discriminate between the different labels as flawlessly as possible (Objective A). The second and third objective integrate the user modality in the parameters of the unimodal classifier. They are both based on the *correlated identity assumption*, as introduced in Section 3.2. According to this assumption, a news article $a_{i}$ and its identity should somehow correlate with the identity of each person $u_{j}\in U_{i}$ spreading the article online (Objective B), and all spreaders $u_{j}\in U_{i}$ should correlate with each other (Objective C).

#### (A) Discriminate between fake and true news.

We use the commonly-used cross-entropy loss on the classifier’s prediction output:

(5) $$L_{pred}=-\overset{N}{\underset{i=1}{\sum }}y_{i}\log P\left(y_{i}\right)+\left(1-y_{i}\right)\log\left(1-P\left(y_{i}\right)\right)$$

with N the number of articles in the training set. By minimising **prediction loss** $L_{pred}$, we force the classifier to not only look for patterns that discriminate between fake and true news but also learn patterns within each class. We provide baselines that are only optimised on this prediction loss.

#### (B) Correlate the article that is evaluated with users who spread it on twitter.

Instead of maximising the correlation between the article and the users, we minimise the cosine distance, as computed in Kocher and Savoy (2017), between their latent representation. The **article–user distance loss** $L_{dist\left(\alpha_{i}\right)}$ first computes the cosine distance between the latent representation $h\left(a_{i}\right)$ of article $a_{i}$ encoded by h, and the latent representation of each $u_{j}\in U_{i}$ encoded by the user encoder, $g\left(u_{j}\right)$. It then takes the arithmetic mean over the S cosine distance results, with S the length of user subset $U_{i}$. If $U_{i}$ is empty, $L_{dist\left(\alpha_{i}\right)}$ is set to 0. This way, the learning algorithm easily handles lacking user information.

(6) $$L_{dist\left(\alpha_{i}\right)}=\left\{\begin{array}{cc} \frac{1}{S}\overset{S}{\underset{j=1,u_{j}\in U_{i}}{\sum }}dist_{cosine}\left(h\left(a_{i}\right),g\left(u_{j}\right)\right)\; & \text{if }U_{i}\neq 0̸\\ 0\; & \text{otherwise} \end{array}\right)$$

The loss over all N training articles is: $L_{dist\left(\alpha \right)}=\sum_{i}^{N}L_{dist\left(\alpha_{i}\right)}$. By optimising the classifier’s parameters on the article–user distance loss, it learns correlations between the identity of the article and those of the users who spread it on Twitter.

#### (C) Correlate users sharing the same article on their Twitter profile.

As for objective B, we minimise the cosine distance instead of maximising correlation. However, we now force the model to reason about the correlation between users sharing the same article. **User–user distance loss** $L_{dist\left(\beta_{i}\right)}$ computes for each $u_{j}\in U_{i}$ the average cosine distance between $g\left(u_{j}\right)$ and the latent representations of the other users $u_{k\neq j}\in U_{i},g\left(u_{k}\right)$, and then takes the arithmetic mean over the S results to obtain a value between 0 and 1. If $U_{i}$ is empty, $L_{dist\left(\beta_{i}\right)}$ is set to 0.

(7) $$L_{dist\left(\beta_{i}\right)}=\left\{\begin{array}{cc} \frac{1}{S}\overset{S}{\underset{j=1}{\sum }}dist\left(u_{j},U_{i}\right)\; & \text{if }U_{i}\neq 0̸\\ 0\; & \text{otherwise} \end{array}\right)$$

(8) $$dist\left(u_{j},U_{i}\right)=\frac{1}{S-1}\overset{S}{\underset{k=1,k\neq j,u_{k}\in U_{i}}{\sum }}dist_{cosine}\left(g\left(u_{j}\right),g\left(u_{k}\right)\right)$$

The loss over all N training articles is: $L_{dist\left(\beta \right)}=\sum_{i}^{N}L_{dist\left(\beta_{i}\right)}$. By optimising the model parameters on the user–user distance loss, the model learns correlations between the users who shared the same news article on Twitter.

#### Combined loss.

The three loss functions - prediction loss $L_{pred}$, article–user distance loss $L_{dist\left(\alpha \right)}$, and user–user distance loss $L_{dist\left(\beta \right)}$ - are combined as a weighted sum:

(9) $$L=\lambda_{1}L_{pred}+\lambda_{2}L_{dist\left(\alpha \right)}+\lambda_{3}L_{dist\left(\beta \right)}$$

where $\lambda_{1}$, $\lambda_{2}$, and $\lambda_{3}$ sum to 1. During training, the parameters of the classifier and the user encoder are optimised with the mean batch loss after each forward pass.

## 4 Problem statement and research questions

### 4.1 Problem statement

The goal of this paper is to design and test a learning algorithm that prevents decision-making based on profiling while still allowing a fake news classifier predicting the veracity of online news article to leverage the social context of those articles. In a multimodal setting, the classifier takes both news article a and user information u and has its parameters optimised using the cross-entropy loss $L_{pred}$ on the prediction output. However, this approach is prone to profiling as it relies on user information during prediction. In this work, classifier f=c∘h only takes a as input. User information u is projected by a parallel encoder g onto the same latent space as a, creating a joint cross-modal latent space $R^{m}$. The parameters of f and g are then optimised using a cross-modal, weighted loss function L. This way, the parameters of f are indirectly guided by u. In contrast to a standard multimodal classifier, user-inspired classifier f cannot profile users when assessing the truthfulness of news articles as it does not have access to any input user information during inference.

### 4.2 Research questions

RQ1 *How does social context impact the performance of a unimodal fake news detection model?*

We compare the prediction performance of f when its parameters are optimised using $L_{pred}$ and when it is constrained using L. We hypothesise that indirectly presenting user information to computational models evaluating the veracity of a given news article improves prediction performance. Not only does it contextualise the news article’s dissemination on Twitter, it also provides insights into the identity of both the news article and its spreaders.

RQ2 *How strongly does model performance rely on the selection of users and tweets?*

$U_{i}$ is represented by a set of user profiles $u_{j}$ which spread news article $a_{i}$ on Twitter, where $u_{j}$ is represented by its profile description $d_{j}$ and/or its user timeline ${tw}_{j}$: $u_{j}=\left[d_{j};{tw}_{j}\right]$. It should be noted that tweets and user profiles disappear or become unavailable over time. It is therefore imperative that f maintains its performance even if certain profiles and tweets are no longer available. In that respect, the effect of user selection $u_{j}\in U_{i}$ and tweet selection for obtaining $tw_{j}$ should be minimal.

RQ3 *Do the models actually find and leverage correlations between articles and users?*

During optimisation, the model parameters of text-based fake news classifier f are optimised by minimising L, which is a weighted combination of three loss functions:

(a) $L_{pred}$; Prediction loss on the prediction output of the classifier;
(b) $L_{dist\left(\alpha \right)}$; Article–user distance loss on the article latent representation yielded by the classifier’s article encoder h and each user latent representation yielded by a parallel user encoder g;
(c) $L_{dist\left(\beta \right)}$; User–user distance loss on all user latent representations yielded by the parallel user encoder g.

By minimising $L_{dist\left(\alpha \right)}$ and $L_{dist\left(\beta \right)}$, the algorithm forces the classifiers to look for correlations between the article and each user ($L_{dist\left(\alpha \right)}$), and among the users ($L_{dist\left(\beta \right)}$). We therefore analyse whether such correlations are found. This is done both in a quantitative and qualitative manner.

RQ4 *To which extent do the enforced cross-modal correlations change the latent space of unimodal fake news classifiers?*

During model training, the latent space $R^{m}$ onto which h projects a and g projects u is coordinated by loss function L. It is thus imperative to investigate how and to which extent $R^{m}$ is influenced and guided by L in our learning algorithm. We visualise $R^{m}$ and measure the overlap between the two prediction classes using statistical dimension reduction techniques. This is done for the baseline setup (i.e., f is optimised using $L_{pred}$) and the setups with our learning algorithm (i.e., f is optimised on L), and analyse their differences.

## 5 Experiments

### 5.1 Data

We evaluate the prediction performance of the three classifiers and the impact of the multimodal learning algorithm using data from different news areas: politics (PolitiFact), entertainment (GossipCop), and COVID-19 (ReCOVery). The first two datasets are part of the larger FakeNewsNet dataset (Shu, Mahudeswaran, Wang, et al., 2020). However, we split them in two separate datasets because they focus on different types of news. An overview of the datasets is given in Table 2. It should be noted that these datasets will become obsolete over time as fake news creators often remove their articles and the profiles with which they spread them from the Internet (Allcott & Gentzkow, 2017).

#### PolitiFact.

The *PolitiFact* dataset comprises fact-checked articles on political news from the PolitiFact^6^ fact-checking website. The articles mainly discuss US politics. Even though the PolitiFact organisation adopts a broader range of fact-check labels on their website, the dataset implements binary labels (i.e., fake or true). In order to extract all article and user features, we use the download script provided by the authors.^7^ After running the script, we can automatically retrieve the title and body text of each article. As for the user features, the script returns the user profiles linked to the tweets that spread the articles on Twitter and retrieves the profile description and 200 latest tweets of each user. In total, 568 complete articles, of which 248 *true* and 320 *fake*, could be automatically extracted.

#### GossipCop.

The second part of the FakeNewsNet dataset consists of fact-checked entertainment news articles extracted from the GossipCop^8^ fact-checking website. On the website, fact-checkers give a score between 0 and 10, with 0 denoting fake and 10 truthful news. As the majority of fact-checked news articles on the GossipCop website appeared to have a score lower than 5, Shu, Mahudeswaran, Wang, et al. (2020) collected articles from the trusted E! Online^9^ website and included them as *true* (1) articles. The low-scoring GossipCop articles are labelled as *fake (0)*. This way, the dataset provides both true and fake news. The data for representing the articles and users are extracted as done for the PolitiFact dataset. In total, 16,963 complete articles, of which 12,904 *true* and 4,059 *fake*, could be automatically crawled.

#### ReCOVery.

(Zhou, Mulay, Ferrara, & Zafarani, 2020). The *ReCOVery* dataset discusses news about COVID-19 and consists of 2,029 news articles about COVID-19. Each article is labelled as either *unreliable* (665 articles) or *reliable* (1,364 articles). We consider the reliability labels synonymous to the true and fake labels used for GossipCop and PolitiFact article. The dataset provides the complete articles (i.e., title and body text). Each article has also been paired with a list of tweet IDs. For each tweet ID, we use the Twitter API to retrieve the profile of the user who spread the tweet and extract their profile description and the 200 latest tweets from their timeline.

**Table 2:** Overview of the three datasets.

Dataset	Domain	True/Reliable	Fake/Unreliable	Total
*PolitiFact*	Politics	248	320	568
*GossipCop*	Entertainment	12,904	4,059	16,963
*ReCOVery*	COVID-19	1,364	665	2,029

Total articles		14,516	5,044	19,560

### 5.2 Experimental setup

Due to the low number of articles in the PolitiFact and ReCOVery datasets, we train and validate the fake news classifiers on all three datasets simultaneously. This way, the models also learn to generalise over a mix of news topics instead of a single domain. We split the datasets in a train (80%), validation (10%), and test (10%) set in a label-stratified manner (random seed = 42).^10^ We report the performance of each fake news classifier for each dataset individually. This allows us to investigate the impact of user integration for each news category. During model training, batch size = 32 when training the CNN and HAN model, while batch size = 8 with the DistilBERT model. We explore four experimental setups:

*base* This setup does not leverage any social context. The classifiers are optimised using the prediction loss on the classification output ($L_{pred}$).
*+d* This setup represents users by their profile description: $u_{j}=\left[d_{j}\right]$. The algorithm takes the classification output and the latent user representations encoded by the separate user encoder and optimises the classifier and the user encoder using the multimodal loss function $L=\lambda_{1}L_{pred}+\lambda_{2}L_{dist\left(\alpha \right)}+\lambda_{3}L_{dist\left(\beta \right)}$.
*+t* This setup represents users by their tweets: $u_{j}=\left[tw_{j}\right]$. The algorithm takes the classification output and the latent user representations encoded by the separate user encoder and optimises the classifier and the user encoder using L.
*+d/t* This setup represents users as a concatenation of their profile description and their tweets: $u_{j}=\left[d_{j};tw_{j}\right]$. The algorithm takes the classification output and the latent user representations encoded by the separate user encoder and optimises the classifier and the user encoder using L.

The Adam optimisation algorithm (learning rate = 1e-4) optimises the parameters after every forward pass. We perform early stopping on the validation loss with patience = 7. To decide on the $\lambda_{1}$, $\lambda_{2}$, and $\lambda_{3}$-values in L for each model, we experiment with different value combinations for CNN${}_{+d}$, HAN${}_{+d}$, and DistilBERT${}_{+d}$. We also test if including both $L_{dist\left(\alpha \right)}$ and $L_{dist\left(\beta \right)}$ increases model performance more than simply including one of the two distance losses. This is done by setting either $\lambda_{2}$ or $\lambda_{3}$ to zero. The different λ-combinations are displayed in Table 3. Based on the validation set, we found the following optimal [$\lambda_{1}$, $\lambda_{2}$, $\lambda_{3}$]-values for each classifier: [0.8, 0.1, 0.1] (CNN), [0.5, 0.25, 0.25] (HAN), [0.33, 0.33, 0.33] (DistilBERT). All models thus benefit from including both distance losses equally.

**Table 3:** Overview of tested λ-values in weighted loss function $L=\lambda_{1}L_{pred}+\lambda_{2}L_{dist\left(\alpha \right)}+\lambda_{3}L_{dist\left(\beta \right)}$ - with $L_{pred}$ the prediction loss, $L_{dist\left(\alpha \right)}$ the article–user distance loss, and $L_{dist\left(\beta \right)}$ the user–user distance loss.

$\lambda_{1}$	$\lambda_{2}$	$\lambda_{3}$	Description
1	0	0	No distance losses (= *base*)
0.5	0	0.5	No article-user distance loss
0.5	0.5	0	No user-user distance loss

0.33	0.33	0.33	All losses equally important

0.5	0.25	0.25	Prediction loss most important,
0.8	0.1	0.1	Distance losses are equally important

0.6	0.3	0.1	Article-user loss more important than user-user loss
0.6	0.1	0.3	User-user loss more important than article-user loss

### 5.3 Results

Given the imbalance between the *fake* and *true* class in the datasets, we report performance results for each label separately and use precision (P), recall (R), and F1-score (F1) as performance metrics.^11^ For sake of brevity, Table 4 displays only the F1-scores. Table 5 goes more in depth and compares the base models with its best performing, user-constrained version in terms of precision, recall, and F1-score. Overall, we observe increased prediction performance when leveraging social context using the learning algorithm. For political news (PolitiFact), representing users by only their profile description during training, ***+d***, yields the highest performance results for CNN and DistilBERT. For entertainment news (GossipCop), adding both description and tweets, ***+d/t***, improves HAN and DistilBERT performance results while CNN again prefers the description-only setting, ***+d***. For COVID-related news (ReCOVery), the user setups that integrate tweets – ***+t*** for CNN; ***+d/t*** for HAN/DistilBERT – outperform the ***base*** and ***+d*** models. Overall, the setups that include a user’s profile description, ***+d*** and ***+d/t***, are the most successful. In terms of competitiveness to previous work, the user-constrained models yield results that are in line with those of other multimodal classifiers on the PolitiFact and GossipCop dataset (Nguyen et al., 2020, Qian et al., 2018, Ruchansky et al., 2017, Shu, et al., 2019). Our profiling-avoiding approach is thus a competitive, more ethical alternative for models that directly rely on user comments (Qian et al., 2018, Ruchansky et al., 2017, Shu, et al., 2019) or profile descriptions (Nguyen et al., 2020) at prediction time. We are, to our knowledge, the first to report multimodal (i.e., news article and users) classification results on the ReCOVery dataset.

**Table 4:** Overview of the performance results (F1-score) for the *fake* and *true* labels. The underlined results indicate that the user-constrained model outperforms its user-unaware base model. The highest results for each model and dataset are marked in **bold**.

***fake***	PolitiFact				GossipCop				ReCOVery			
	*base*	*+d*	*+t*	*+d/t*	*base*	*+d*	*+t*	*+d/t*	*base*	*+d*	*+t*	*+d/t*
CNN	.4681	**.5600**	.5490	.5200	.6531	**.6542**	.6487	.6484	.6549	.6783	**.7368**	.6964
HAN	**.7541**	.7000	.7000	.7213	.6592	.6555	.6555	**.6610**	.7826	.7794	.7794	**.7941**
DistilBERT	.7333	**.7368**	.5926	.6897	.6444	.6369	.6108	**.6731**	.7519	.6261	.6667	**.7805**

***true***	*base*	*+d*	*+t*	*+d/t*	*base*	*+d*	*+t*	*+d/t*	*base*	*+d*	*+t*	*+d/t*

CNN	.6377	**.6667**	.6462	.6364	**.9118**	.9113	.9079	.9098	.8632	.8693	**.8944**	.8811
HAN	**.7273**	.6786	.6786	.6909	.8950	**.8978**	**.8978**	.8928	.8846	.8855	.8855	**.8931**
DistilBERT	.7143	**.7457**	.6452	.6897	.9096	.9106	.9048	**.9120**	.8755	.8481	.8456	**.9018**

**Table 5:** Comparison of baseline (base) against best performing user setup (*+d*, *+t*, *+d/t*) for each model and dataset. We abbreviate DistilBERT as DBERT in this table. For simplicity, we take the same user setup for both labels (fake/true). Improvements over *base* models are underlined.

(a) PolitiFact				
***fake***		P	R	F1
CNN	base	.7857	.3333	.4681
	*+d*	.8235	.4242	.5600

HAN	base	.8214	.6970	.7541
	*+d/t*	.7857	.6667	.7213

DBERT	base	.8148	.6667	.7333
	*+d*	.8750	.6364	.7368

***true***		P	R	F1

CNN	base	.5000	.8800	.6377
	*+d*	.5366	.8800	.6667

HAN	base	.6667	.8000	.7273
	*+d/t*	.6333	.7600	.6909

DBERT	base	.6452	.8000	.7143
	*+d*	.6471	.8800	.7457

(b) GossipCop				
***fake***		P	R	F1
CNN	base	.7951	.5542	.6531
	*+d*	.7882	.5591	.6542

HAN	base	.6684	.6502	.6592
	*+d/t*	.6569	.6650	.6610

DBERT	base	.7845	.5468	.6444
	*+d/t*	.7724	.5936	.6731

***true***		P	R	F1

CNN	base	.8722	.9552	.9118
	*+d*	.8732	.9529	.9113

HAN	base	.8921	.8988	.8950
	*+d/t*	.8945	.8910	.8928

DBERT	base	.8701	.9529	.9096
	*+d/t*	.8811	.9451	.9120

(c) ReCOVery				
***fake***		P	R	F1
CNN	base	.7872	.5606	.6549
	*+t*	.8750	.6364	.7368

HAN	base	.7500	.8182	.7826
	*+d/t*	.7714	.8182	.7941

DBERT	base	.7463	.7576	.7519
	*+d/t*	.8421	.7273	.7805

***true***		P	R	F1

CNN	base	.8092	.9248	.8632
	*+t*	.8411	.9549	.8944

HAN	base	.9055	.8647	.8846
	*+d/t*	.9070	.8797	.8931

DBERT	base	.8788	.8722	.8755
	*+d/t*	.8732	.9323	.9018

## 6 Discussion

We investigate the four research questions we introduced in Section 4:

RQ1 *How does user knowledge impact the performance of a unimodal fake news detection model?* (Section 6.1)
RQ2 *How strongly does model performance rely on the selection of users and tweets?* (Section 6.2)
RQ3 *Do the models actually find and leverage correlations between articles and users?* (Section 6.3)
RQ4 *To which extent do the enforced cross-modal correlations change the latent space of the classifiers?* (Section 6.4)

We then elaborate on the ethical principles of fake news detection and AI systems in general, and indicate how our learning algorithm mitigates violations of those principles (Section 6.5). A brief overview of possible implications of this work concludes the discussion (Section 7).

### 6.1 Impact of users on model performance

#### Prediction confidence.

If we take classification performance as the only impact measure, the learning algorithm is then shown to have a greater impact on political and COVID-19 news articles than on entertainment-related news. Note that the adopted training approach where the classifiers were trained on all datasets simultaneously might have encouraged the base model to learn better discriminating feature representations of gossip news than of political and COVID-related news. As a result, the impact of our learning algorithm seems marginal when assessing gossip. As prediction performance is not the only way to measure impact, we also evaluate our learning algorithm’s impact on the prediction confidence of the classifiers. We do this by comparing the prediction probabilities $P\left(y_{i}\right)$ yielded by the baseline model and its user-constrained counterparts (+d, +t, +d/t). For our analysis, we focus on entertainment articles as the GossipCop dataset is sufficiently large for statistically testing. We mimic a model’s ultimate decisions and take for each article $a_{i}$ the label with the highest prediction probability as final label. Hence, the probabilities in our analysis lie between 0.5 and 1. We rely on the two-sample T-test and Kruskal–Wallis H-test for statistical testing. Overall, we observe high levels of confidence across all models. Considering the DistilBERT model, the two statistical tests both confirm that the model constrained on users represented by their profile description and tweets (i.e., DistilBERT${}_{+d/t}$) returns significantly higher prediction probabilities than its baseline: DistilBERT${}_{base}$ (μ = 0.861697, $\sigma^{2}$ = 0.018571) and DistilBERT${}_{+d/t}$ (μ = 0.891369, $\sigma^{2}$ = 0.015190)[T = −6.658362, p<0.01; H = 33.668810, p<0.01]. When leveraging tweets-only user representations, the DistilBERT${}_{+t}$ model is not as confident as the baseline: DistilBERT${}_{base}$ (μ = 0.861697, $\sigma^{2}$ = 0.018571) and DistilBERT${}_{+t}$ (μ = 0.847578, $\sigma^{2}$ = 0.014585) [T = 3.196966, p<0.01; H = 64.073104, p<0.01]. Only the Kruskal–Wallis H-test rejects the null hypothesis for DistilBERT${}_{base}$ (μ = 0.861697, $\sigma^{2}$ = 0.018571) and DistilBERT${}_{+d}$ (μ = 0.859106, $\sigma^{2}$ = 0.014207) [H = 23.783539, p<0.01]. The statistical tests confirm the same for the HAN model: HAN${}_{+d/t}$ is more confident than the base model. The tests are inconclusive for the CNN model. In all, the confidence analysis shows that *the user-constrained DistilBERT and HAN models are more confident about their predictions than their baseline when users are represented by both their profile description and tweets*.

#### Error analysis.

We continue by performing an error analysis on the predicted labels for the test set. More specifically, we investigate whether the base and user-constrained models make the same mistakes. For sake of brevity, Table 6 only displays the aggregated results over all three datasets. Overall, *the user-constrained models do not consistently make the same mistakes as the base models*. This is most notable for DistilBERT as about 13% of the articles across all datasets are differently classified by the user-constrained models than the base model. This difference is less distinct for CNN and HAN; 4% and 8%, respectively. Upon investigating the user settings individually, we observe that including profile descriptions in the user representations (i.e., +d and +d/t) yields slightly more predictions that are different from those of the base models than when descriptions are excluded (i.e., +t). Moreover, each user setting corrects previous mistakes and makes new ones that the other setups do not. This is shown by the considerably lower number of articles for which all three user-constrained models predict a label that is different from that predicted by the base model. For DistilBERT, for example, merely 21 of the 144 new prediction errors are made by in all three user settings. Regarding articles that are incorrectly predicted by the base and user-constrained models, we could not identify striking signals in either the article or the user profiles to which prediction errors could be attributed. Although we observe that the HAN models tend to attend to person entities when encoding news articles, the models do not consistently assign the same prediction label to popular entities such as ‘Trump’ and ‘Kardashian’ in the datasets. Later in the discussion, we investigate whether certain correlations lead to erroneous prediction when we manually analyse the correlations in the texts of the article and its spreaders’ profiles (Section 6.3).

**Table 6:**
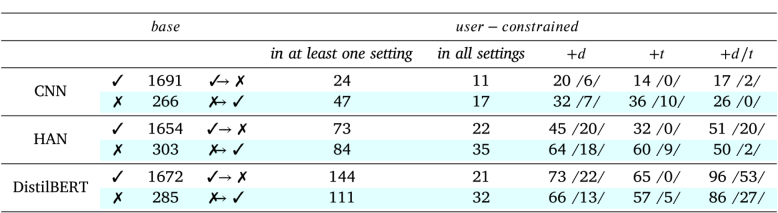
Error analysis on aggregated test results over all three datasets. The table displays the absolute number of articles for which the *base* model predicts an correct label (✓) while its *user-constrained* counterparts predict an incorrect label (✓$\overset{}{⟶}$ ✗), and vice versa (✗, ✗$\overset{}{⟶}$ ✓, in blue). For each user setting (+d, +t, +d/t), the number within the slashes (/ /) indicates the number of articles for which that particular setting is the only one predicting a different label.

### 6.2 User and tweet selection

**Fig. 6:**
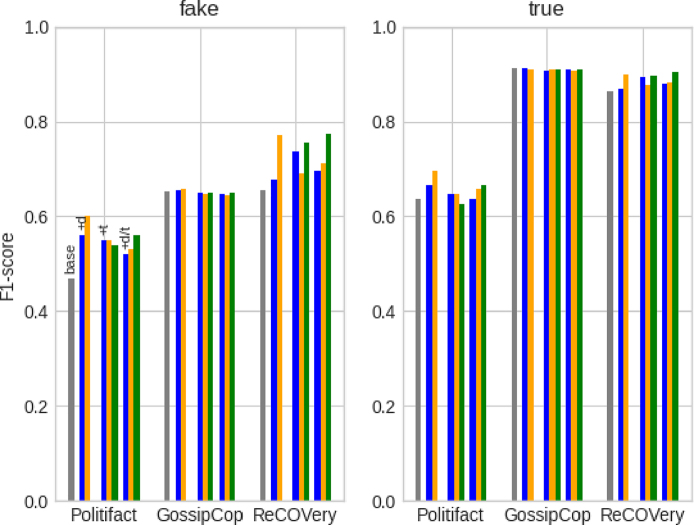
CNN performance (F1-score) when changing the user selection method from early to late dissemination (in orange) and when changing the tweet selection from most recent to oldest (in green). The original baseline results are displayed in grey (CNN${}_{base}$) and the original user-constrained results in blue (CNN${}_{+d}$, CNN${}_{+t}$, CNN${}_{+d/t}$) . (For interpretation of the references to colour in this figure legend, the reader is referred to the web version of this article.)

The default learning algorithm takes an article’s early spreaders and represents them by their most recent tweets. We therefore investigate how user and tweet selection impacts model performance. We start with user selection. As user profiles can disappear over time and might be no longer retrievable, model performance should not rely on the composition of an article’s user subset too heavily. In the original experiments, each user subset $U_{i}\subseteq U$ reflects an article’s *early* dissemination audience as we took the user IDs linked to the lowest tweet IDs in the article’s tweet list. We now investigate how its *late* dissemination audience impacts model performance. Instead of the *lowest*, we select the user IDs linked to the *highest* tweet IDs in the tweet lists. These new user subsets are then used to optimise the models. By presenting more recent spreaders during model optimisation, the models may find different article–user and user–user correlations. We expect though that the early spreaders display higher correlations to the article and subsequently influence the user-constrained models more positively, as the creator of the article is likely the first to share it on Twitter. For brevity, Fig. 6 only displays the performance results of the CNN model (in orange), but we discuss results for all three classifiers. Although we conjectured that early spreaders are probably more correlated to the article, the early dissemination audience does not perform consistently higher than the late dissemination audience. On the ReCOVery dataset, the latter even outperforms all ***base*** models on all metrics with all user setups, while this was only the case for CNN (all setups), HAN (***+t***), and DistilBERT (***+d/t***) in the original experiments. These positive results indicate that *models do not need to look at the characteristics of an article’s early dissemination audience to achieve higher performance.*

Next, we examine the impact of tweet selection. In the original experiments, the ***+t*** and ***+d/t*** setups represented users by their most recent tweets. We randomly extract 10,000 tweet timelines from the user subsets and investigate their time spans. It appears that the time span of more than half of the timelines do not exceed three months while less than one in four timelines span more than a year. This means that the user identities contained in the original representations were fairly recent. When we would take the *oldest* tweets from the users’ timelines instead, the user representations may reflect slightly older, perhaps different user identities. Given the articles’ publication dates (i.e., PolitiFact: 2008–2018, GossipCop: 2017–2018, ReCOVery: 2020), we expect increased model performance for ReCOVery articles because a user’s oldest tweets could have been posted around the same time as the article and thus reflect that user’s identity at spreading time. Fig. 6 only displays the impact on CNN prediction for sake of brevity (in green). Overall, the alternative tweet selection does not impact CNN and HAN performance more than the original selection. The user-constrained DistilBERT models, on the other hand, now outperform the baseline on all three datasets with both user setups. For comparison, the original experiments only led to above-baseline DistilBERT results on PolitiFact and ReCOVery data with the ***+d/t*** setup. We do not, however, observe the conjectured, across-model positive impact on ReCOVery prediction. These results indicate that *the success of our multimodal learning algorithm does not heavily depend on the selection of tweets to represent an article’s spreaders*. Nevertheless, the impact of leveraging the user identities at spreading time is still unclear as most user timelines in the user subsets do not have a wide enough time span to explore this.

### 6.3 Correlations

**Fig. 7:**
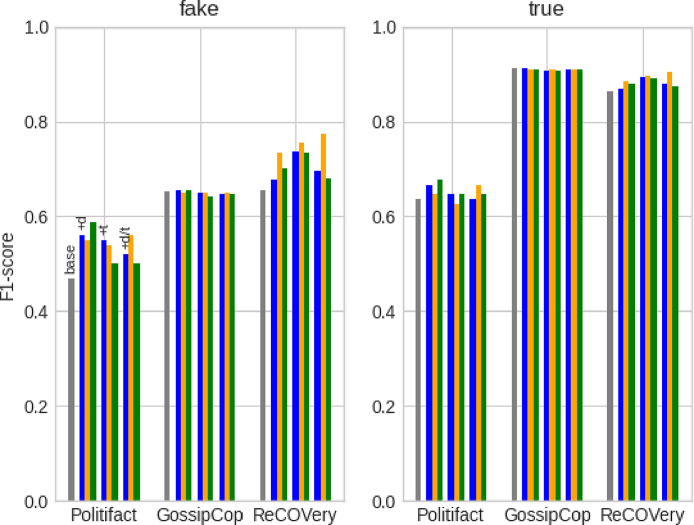
CNN performance (F1-score) when distorting the correlations between articles and users by randomly assigning the user subsets to the articles (in orange) and additionally distorting the correlations between the users by randomly assigning a user to a user subset. The original baseline results are displayed in grey (CNN${}_{base}$) and the original user-constrained results in blue (CNN${}_{+d}$, CNN${}_{+t}$, CNN${}_{+d/t}$) . (For interpretation of the references to colour in this figure legend, the reader is referred to the web version of this article.)

In Section 3.2, we formulated the correlated identity assumption, which states that there exist correlations between the identity of an article and those of its spreaders (article–user correlation), and between the spreaders as a group (user–user correlation). The user-inspired learning algorithm enforced those two correlations using $L_{dist\left(\alpha \right)}$ and $L_{dist\left(\beta \right)}$, respectively, when optimising three text-based fake news classifiers. We now investigate if the user-constrained models actually detected and leveraged article–user and user–user correlations, or if the increased performance simply resulted from leveraging more data. We evaluate this in a quantitative and qualitative manner. We start by distorting possible correlations between articles and users (*Random Subset*). For this, we randomly pair article $a_{i}$ with a user subset $U_{k\neq i}$ (random seed = 42). Note that we do not change the composition of the subsets. While we maintain possible correlations between the users in the first distortion experiment, we now change not only the correlations between the articles and users, but also the correlation between the users (*Random Subset + Composition*). We obtain distorted user subsets by randomly grouping users in subsets (random seed = 42): $U_{i,r}\subseteq U,U_{i,r}=\left\{u_{j}|u_{j}\notin U_{i}\right\}\wedge \left|U_{i,r}\right|=\left|U_{i}\right|$. For brevity, Fig. 7 only reports distortion results for the CNN classifier but we discuss results for all three models. The less distorting, first experiment performs overall better than the more distorting, second experiment. However, they both counter-intuitively increase results on the ReCOVery dataset for all models — independent of the user setup. They often even outperform the user-constrained models from the original experiments.

**Fig. 8:**
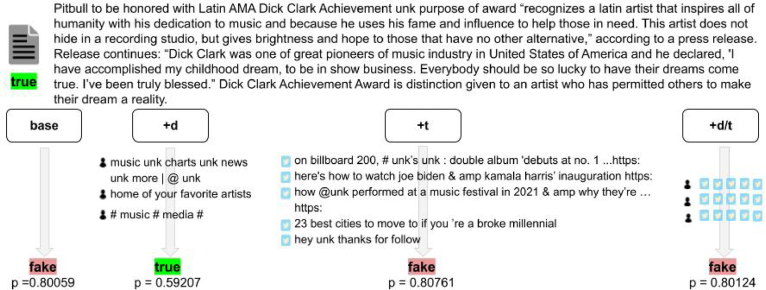
Example taken from the GossipCop training set for which the base and user-constrained HAN models disagree on the veracity label.

Given the counter-intuitive results of our distortion experiments, we now evaluate if we can manually recognise correlations between the text of the article and the textual content generated by its spreaders. We also explore whether those correlations guide the classifier towards the ground-truth label. We obtain a set of randomly selected news articles with their associated user subsets from the GossipCop dataset (random seed = 42) and pair them with the predicted label yielded by the base and user-constrained HAN models. We specifically focus on cases where the user-constrained models and the base model disagree on the veracity label as these may reveal how user insights lead to different predictions. Fig. 8 presents such a case. Overall, the qualitative analysis does not reveal clear and consistent correlation patterns between news articles and user-generated texts that might influence model prediction. Despite the positive effect of the correlated identity assumption on model performance, the results suggest that *the models benefit from enforcing cross-modal and unimodal correlations without actually uncovering the assumed user-related correlations as stated in the correlated identity assumption*. This opens ample opportunities for further research to explore and enforce different assumptions on the relations between social media users and the external content they share.

### 6.4 Guided latent space

Our learning algorithm inspires the latent space of a text-based classifier with user insights by constraining it with a weighted combination of three loss functions: prediction loss $L_{pred}$, article–user distance loss $L_{dist\left(\alpha \right)}$, and user–user distance loss $L_{dist\left(\beta \right)}$. We provided baselines that were only optimised on the prediction loss. This allows us to investigate how our user-inspired approach guided the latent space of the text-based classifiers.

We use some established exploratory data analysis techniques for dimension reduction and data visualisation to appreciate the effect of the latent space choice in our experiments. We illustrate here approaches based on principal component analysis (PCA, Hotelling, 1936) and multidimensional scaling (Cox & Cox, 1994). The first is to capture the information hidden in the many data dimensions with a change of basis of the data. The second is to visualise the level of similarity among the data rows through the pairwise distances that we have calculated. Note that in this work we apply a robust variant of the methods; this way, we avoid that present outliers and deviations from canonical distributional assumptions distort the results.^12^ We strongly believe that this precautionary measure, often neglected, is essential in the analysis of so complex data (for details, see Hubert et al., 2005, Hubert et al., 2009). Before illustrating the application of the two methods to our case, we give an informal introduction of their original (non-robust) version, which helps interpreting correctly the outcomes.

**Fig. 9:**
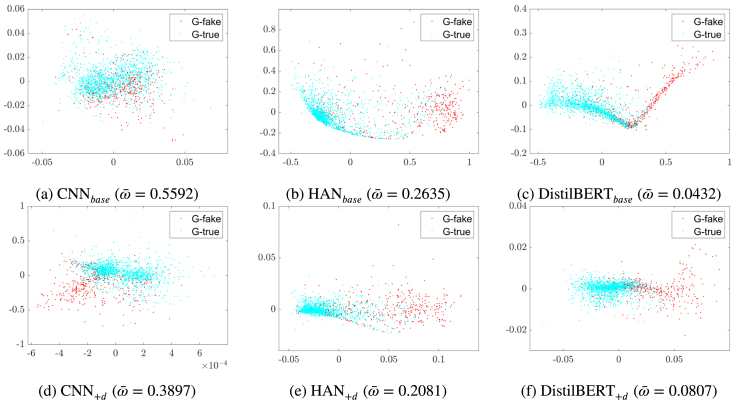
The panels visualise the latent representations of all GossipCop articles in the *test set*. The visualisations are generated using **multidimensional scaling** based on cosine similarity, where $\overset{¯}{\omega }$ quantifies the average overlap between the ground-truth fake class (in red) and true class (in blue).(For interpretation of the references to colour in this figure legend, the reader is referred to the web version of this article.)

**Fig. 10:**
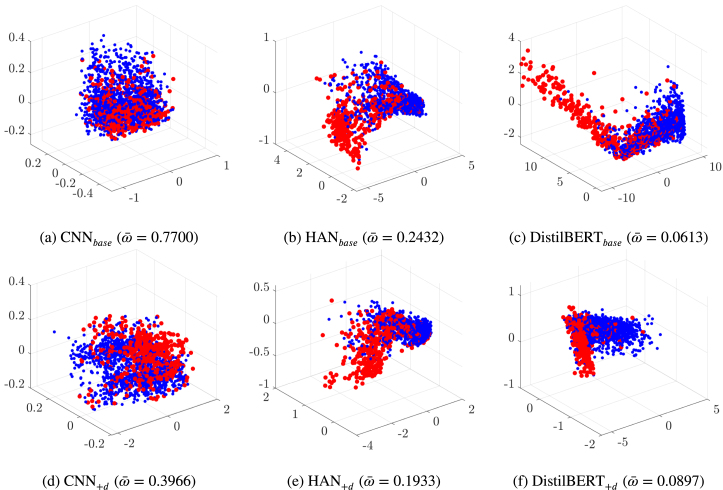
The panels visualise the latent representations of all GossipCop articles in the *validation set*. The visualisations are generated using the scores of the three main **principle components**, where $\overset{¯}{\omega }$ quantifies the average overlap between the ground-truth fake class (in red) and true class (in blue).(For interpretation of the references to colour in this figure legend, the reader is referred to the web version of this article.)

The objective of PCA is to replace the initial p quantitative variables – say $X_{1},X_{2},\ldots ,X_{p}$ – that can be correlated, with a new set of variables – the principle components $Y_{1},Y_{2},\ldots ,Y_{q}$ – which have these properties: (i) they are orthogonal, that is, they are not correlated; (ii) they are in decreasing order with respect to their variance. The first principle component $Y_{1}$ is the linear combination of the p initial variables having maximum variance. The second, $Y_{2}$, is the linear combination of the p variables with the variance immediately lower of the variance of $Y_{1}$; in addition, $Y_{2}$ is not correlated to $Y_{1}$. The process can continue till q=p. However, if the p original variables are very correlated, a number q<p of principle components will take into account a high percentage of the total variance, that is, the first q components will give a good approximation in (much) lower dimension of the structure of the data. In summary, the q latent variables are obtained with a linear transformation that projects the original variables in a new Cartesian space with the variable $Y_{1}$ in the first axis, $Y_{2}$ in the second axis, and so on. Fig. 10 shows the first q=3 components in the axes, which already capture more that 75% of the total variance.

Multidimensional scaling approaches the goal of projecting data into a lower-dimensional manifold in a different way (complementary, in our case). It starts with some measure of distance between each pair of data units $u_{i}$ and $u_{j}$ ($\in R^{p}$), for example the cosine similarity $d_{ij}=dist_{cosine}\left(u_{i},u_{j}\right)$. The distances are stored in a n×n similarity matrix D, or its upper triangle form if distances are symmetric. Then, the method returns a configuration of n points (rows) in q<p dimensions (columns), so that the Euclidean distances between these points approximate a monotonic transformation of the corresponding similarities in D. For example, the default least-squares scaling method seeks values $z_{1},z_{2},\ldots ,z_{n}\left(\in R^{q}\right)$ that minimise a *stress function* in the formulation of Kruskal–Shephard (Shepard, 1980), ${\left[\sum_{i\neq j}{\left(d_{ij}-\|u_{i}-u_{j}\|\right)}^{2}\right]}^{1/2}$, using an iterative algorithm that can be chosen to be statistically robust to the presence of noisy or outlying similarity values by applying classical Huber’s weighting in the estimation. Note that the formula, like other options in the literature, does not depend on the original data units: it is sufficient to dispose of the pairwise similarities between the units to compute it. The idea is that the units $z_{i}$ in the lower dimension $R^{q}$ preserve the pairwise distances of D as much as possible.

Let us now test the robust version of the two approaches on the GossipCop dataset, which is adequately large for statistical testing. Fig. 9 shows the multidimensional scaling visualisations of all models for the GossipCop test set. The panels display a two-dimensional representation of the n rows of the latent space, where the Euclidean distances between them translate the information about the pairwise similarities in the Cartesian space. Fig. 10 shows the three main principle components for the GossipCop validation set. The two types of visualisation clearly show that our learning algorithm forced the classifiers to separate more between fake and true news in their latent space. This separation is quantified with an overlap measure^13^ ($\overset{¯}{\omega }$) which expresses the probability of misclassification assuming a Gaussian mixture model as data generating process (Maitra & Melnykov, 2010). Note that the overlap measure is quite reliable in the representations of Fig. 10, where the Gaussian mixture is clearly appropriate. The multidimensional scaling generates groups that are rather skewed in some cases, which might bias the proposed separation estimate. This suggests that robust PCA should be preferred in view to run an extensive assessment of our approach, which is a possible follow up of this work. We can conclude that *our user-inspired learning algorithm encouraged the text-based fake news classifiers to learn latent representations that better discriminate between true and fake articles*.

Note that in analysing the GossipCop dataset with these dimension reduction methods we also apply additional monitoring features that facilitate selecting key parameters linked to the model and the estimation procedure. In particular, given that the robust version of PCA removes an appropriate percentage of deviations and outliers, the monitoring feature is also used to study the fine-grained structure of the data and appreciate the effect of the outliers (Riani et al., 2012, Torti et al., 2021). This *monitoring approach* is receiving increasing attention in the statistical literature (see also Cappozzo, García Escudero, Greselin, & Mayo-Iscar, 2021 for example) but its application to high-dimensional data – which is just approached here – is new. The MATLAB script and a representative data sample used to make this part of the data analysis is available as an example of the FSDA toolbox,^14^ a comprehensive statistical package for robust data analysis.

Regarding the statistically robust method we used to analyse and visualise the impact of user knowledge on the classifiers’ latent space, we argue that it has wider potential within the machine learning field. For example, it could be used to understand and perhaps clean the fine-grained structure of training and validation data. This way, one can remove possible disturbances originating from the data in the model estimates and improve generalisation performances. We also argue that all these potential extensions should be better addressed in a framework where data grouping is done simultaneously in the rows and columns of the data matrix. The framework is known under different names, block-clustering in Govaert and Nadif (2008) but also bi-clustering or co-clustering in other works. This change of perspective would allow to conduct in a principled way dimension reduction and clustering in parallel, rather than in tandem (which is what we have done in these first experiments).

### 6.5 On the ethics of user-reliant fake news detection

Using user information to detect fake news entails a number of ethical implications. We discuss these using the Ethics Guidelines for Trustworthy AI mandated and published by the European Commission (High-Level Expert Group on AI, 2019). The guidelines state that artificial intelligence is trustworthy when it is lawful, ethical, and robust. As this paper focuses on the ethics side of AI, we elaborate on four ethical principles and the fundamental rights they are based on. We direct interested readers to the guidelines for further discussion on lawfulness and robustness. Considering a person’s fundamental rights, computational systems are expected to respect a person’s dignity, their individual freedom, and their rights as citizens. They should also refrain from excluding and discriminating any individual or group. Based on those rights, trustworthy AI should strive to adhere to the following four ethical principles (High-Level Expert Group on AI, 2019):

(i) Respect for human autonomy
(ii) Prevention of harm
(iii) Explicability
(iv) Fairness

Respect for human autonomy and prevention of harm entail that AI systems should enrich people’s abilities to perform tasks and further strengthen their connections with others while safeguarding their dignity and mental/physical integrity. Systems identifying and signalling possibly deceptive news assist their users in distinguishing credible and accurate information from intentionally fake news. From the perspective of those users, it could thus be argued that fake news detectors are – to a certain extent – ethical AI systems. However, computers – just like humans – are not flawless. A computational system often fails to recognise fake information while it unjustly regards truthful news as fake. The latter is especially harmful because the system not only misinforms its users but also discredits the news article and, indirectly, its author. That negative impact is more direct and harmful when the decision process of an AI system also relies on user information from news authors and social media users. Incorrect predictions could malign an individual’s integrity and credibility, thus infringing the ethical principle of prevention of harm. Even if the detector correctly separates true from fake, it does not necessarily mean that the social media users linked to the detected fake news are aware of its falsity or have malicious intentions. We therefore argue that a system should refrain from predicting whether or not an individual is a fake news spreader because intentions and knowledge need to be taken into consideration. It is one thing to detect and identify content as fake news, but another to label actual people. Especially when the detector does not disclose why and how it decided on that label (ethical principle of explicability); a decision could be based on spurious relations and indications in a user’s representation in the model. Moreover, a fake news detector could start to rely too heavily on user profiling or almost completely ignore the article input. AI developers must therefore ensure that detection models do not overly depend on user input. This relates to the ethical principle of fairness, which states that an AI system should not bias, discriminate, or stigmatise individuals and groups. Fake news detectors could violate this ethical principle when users are unjustly profiled based on their characteristics, their comments, or their sharing history. This kind of model bias is often the result of biased training data.

This paper introduced a novel user integration method to avoid decision making based on profiling in user-informed fake news detection. However, other essential characteristics of ethical, trustworthy AI systems such as model explainability still need to be addressed. We therefore argue that our approach should not be regarded as fully ethical yet but rather as a substantial step towards ethical fake news detection.

## 7 Implications

The implications of this work are mainly situated in the field of ethical AI. Firstly, this work may affect the way ethical fake news detection is approached technically. We showed that a profiling-avoiding algorithm does not need to ignore available user information or artificially alter user representations using debiasing techniques. Instead, an algorithm can still benefit from the user modality without residing to profiling by integrating user data using a cross-modal objective function. Other assumptions on the relation between a news article and its audience can be formulated, tested, and assessed. This way, AI systems are guided by real-world knowledge and phenomenons when making sense of news articles and their characteristics. Our work furthermore attributes to the societal impact of automated fake news detection. Apart from respecting human autonomy by providing a system that draws attention to the falsity and deceptiveness of information, our profiling-avoiding algorithm also adheres to ethical principles of fairness (i.e., people should not be discriminated or stigmatised) and prevention of harm (i.e., people’s integrity and credibility should not be maligned). These are principles that the European Commission highlighted in their guidelines for trustworthy AI (High-Level Expert Group on AI, 2019).

Lastly, the implications of this work stretch further than the fake news detection task. It may have important technical implications for the way user information is integrated in neural classifiers overall. The most popular way to present user data to neural classifiers is by giving it as input to the model. The model then reasons over the user modality when deciding on a classification label. We, however, deviated from that classic approach and integrated user knowledge by giving it as input to the objective function used to constrain the model parameters. This way, the user modality is no longer part of the model architecture, allowing for easy data handling when user information is missing.

## 8 Conclusion

This paper addressed the unethical nature of profiling-dependent decision-making in the fake news detection task and introduced a novel method for detection models to avoid profiling while still leveraging the rich insights on social context held by social media users. We took inspiration from the social sciences and formalised a correlated identity assumption which served as the user integration method in our multimodal learning algorithm. In our experiments, the algorithm *inspired* three text-based classifiers with user knowledge and context using a cross-modal loss function during model optimisation. The increased prediction performance of the user-constrained classifiers tells us that systems and people enhance each other — even if user information is not directly available. While fake news classifiers facilitate the rapid detection of deceptive content on social media and support people in their information processing, people help systems to contextualise given input and consequently improve their detection performance. Furthermore, statistical visualisation techniques showed that guiding computational systems using insights from the social sciences on human behaviour and identity positively impacts the way classifiers model fake and true news articles. This paves the way for further research to build on our user-inspired learning algorithm and test other cross-modal, interdisciplinary assumptions in tasks related to fake news detection or, more broadly, in any task requiring social context. In all, ethical AI is a challenging goal and profiling avoidance is just one of the many points on its checklist. Nevertheless, we believe that is an essential step that requires more attention and thorough investigation.

## CRediT authorship contribution statement

**Liesbeth Allein:** Conceptualization, Methodology, Software, Validation, Formal analysis, Investigation, Data curation, Writing – original draft, Visualization. **Marie-Francine Moens:** Writing – review & editing, Supervision, Conceptualization. **Domenico Perrotta:** Formal analysis, Writing – review & editing, Visualization, Supervision, Project administration, Funding acquisition.

## Declaration of Competing Interest

The authors declare that they have no known competing financial interests or personal relationships that could have appeared to influence the work reported in this paper.

## Data Availability

Data will be made available on request.

## Footnotes

1 See https://eur-lex.europa.eu/legal-content/EN/TXT/?uri=celex%3A32016R0679; Recital 71.

3 The articles are tokenized using the tokenizer from the NLTK toolkit (Bird, Klein, & Loper, 2009); we initialise the word embedding layer with GloVe embeddings pretrained on Wikipedia 2014 and Gigaword 5 (uncased, 6B vocabulary of 400k unique tokens).

4 In case of article encoding, the model regards a title as one sentence. In case of user encoding, profile descriptions and tweets are each regarded as one sentence.

5 DistilBERT has been shown to achieve similar performance on several prediction tasks as its undistilled parent, BERT, while having 40% fewer parameters (Sanh et al., 2019). We implement the ‘distilbert-base-uncased’ pretrained DistilBERT model and tokenizer from the Huggingface Transformers library (Wolf, et al., 2020).

6 https://www.politifact.com/.

7 https://github.com/KaiDMML/FakeNewsNet.

8 http://www.gossipcop.com/.

9 https://www.eonline.com/.

10 Data splits can be found here: https://github.com/laallein/preventing_profiling_ethical_FND.

11 Computed using Pedregosa, et al. (2011).

12 We apply the robpca function of the LIBRA toolbox (Verboven & Hubert, 2010) and the MATLAB function mdscale with option statset (‘Robust’,‘on’, ‘RobustWgtFun’,‘huber’) (= Huber’s weighting) for the robust PCA and multidimensional scaling, respectively.

13 We use the FSDA overlap function as described in Riani, Cerioli, Perrotta, and Torti (2015).

14 The toolbox with full examples can be downloaded from https://github.com/UniprJRC/FSDA; the specific case is part of script examples_ multivariate.m.

## Footnotes

2 As model performance scarcely increased with a higher S, we keep a lower S for sake of memory usage.
